# Predicting the severity of postoperative scars using artificial intelligence based on images and clinical data

**DOI:** 10.1038/s41598-023-40395-z

**Published:** 2023-08-18

**Authors:** Jemin Kim, Inrok Oh, Yun Na Lee, Joo Hee Lee, Young In Lee, Jihee Kim, Ju Hee Lee

**Affiliations:** 1 Department of Dermatology, Yongin Severance Hospital, Yonsei University College of Medicine, Yongin-si, Gyeonggi-do South Korea; 2 Scar Laser and Plastic Surgery Center, Yonsei Cancer Hospital, Yonsei University College of Medicine, Seoul, South Korea; 3 LG Chem Ltd., Seoul, South Korea; 4 Department of Dermatology and Cutaneous Biology Research Institute, Severance Hospital, Yonsei University College of Medicine, Seoul, South Korea

**Keywords:** Machine learning, Predictive medicine, Risk factors

## Abstract

Evaluation of scar severity is crucial for determining proper treatment modalities; however, there is no gold standard for assessing scars. This study aimed to develop and evaluate an artificial intelligence model using images and clinical data to predict the severity of postoperative scars. Deep neural network models were trained and validated using images and clinical data from 1283 patients (main dataset: 1043; external dataset: 240) with post-thyroidectomy scars. Additionally, the performance of the model was tested against 16 dermatologists. In the internal test set, the area under the receiver operating characteristic curve (ROC-AUC) of the image-based model was 0.931 (95% confidence interval 0.910‒0.949), which increased to 0.938 (0.916‒0.955) when combined with clinical data. In the external test set, the ROC-AUC of the image-based and combined prediction models were 0.896 (0.874‒0.916) and 0.912 (0.892‒0.932), respectively. In addition, the performance of the tested algorithm with images from the internal test set was comparable with that of 16 dermatologists. This study revealed that a deep neural network model derived from image and clinical data could predict the severity of postoperative scars. The proposed model may be utilized in clinical practice for scar management, especially for determining severity and treatment initiation.

## Introduction

Scarring is a common medical problem that affects patients cosmetically and can cause functional impairment and psychosocial burdens. Hypertrophic scars and keloids frequently develop after surgical procedures. The incidence of hypertrophic scars after a surgical procedure is estimated to be 40‒70% without adequate management^1^, and they can significantly impair quality of life^2^. Post-thyroidectomy scars are particularly problematic because of their location (exposed area of the neck), the relatively young age of the affected patients, and the rapidly increasing incidence of thyroid cancer^3^. Furthermore, since the underlying molecular mechanism of wound healing and scar formation is complex^4^, the predisposing factors or prognostic markers for hypertrophic scarring are also not completely understood^5^. Regarding post-thyroidectomy scars, several clinical risk factors related to hypertrophic scarring have been reported, such as young age, high body mass index (BMI), scar-related symptoms, incision site near the sternal notch, prominent sternocleidomastoid muscles, and a history of abnormal wound healing or pathologic scarring^3,5,6^.

In the era of artificial intelligence (AI), convolutional neural networks (CNN) have been successfully introduced, forming the basis for various emerging applications in dermatology^7^. Current studies using CNN in dermatology have mainly focused on classifying skin diseases, especially skin cancers^8–11^, or lesion identification and quantification via segmentation algorithms^12,13^. However, recent radiology studies have revealed that implementing a deep learning model that combines imaging and clinical data can predict disease severity, risk of progression, and treatment response^14–16^.

Therefore, we aimed to develop an AI model that could predict the severity of postoperative scars using medical images and clinical data. Furthermore, we compared the performance of the AI model with that of dermatologists.

## Materials and methods

### Study design and participants

We performed a retrospective study and identified patients with post-thyroidectomy scars who presented to the Scar Laser and Plastic Surgery Center in the Yonsei Cancer Hospital, Seoul, Republic of Korea, from September 2015 to December 2021. The investigation conforms with the principles outlined in the Declaration of Helsinki and ethical principles for human research. The Institutional Review Board of Yonsei University Severance Hospital approved the research protocols including any relevant details in the method section in this manuscript (approval number 4-2022-0741). Also, informed consent from the study subjects was waived by the Institutional Review Board of Yonsei University Severance Hospital due to the retrospective study design. However, specific consent was obtained from the patient, who agreed to publish their clinical image as a figure in an online open-access publication. For inclusion in the study, we considered all patients who were referred to the dermatology clinic for scar minimization treatment after thyroidectomy procedures, such as conventional thyroidectomy, minimally invasive thyroidectomy (MIT), modified radical neck dissection (MRND), or transaxillary robotic thyroidectomy. Patients were excluded if medical images of the scar site were not captured during their clinic visit. Furthermore, even if photos were taken, patients were excluded if the quality of these images was compromised to such an extent (due to blurring or other factors) that it was difficult to discern the scars.

In the main dataset, we randomly assigned patients to the model training, validation, and internal testing datasets (7:1:2). We also independently collected data on post-thyroidectomy patients who presented to the Department of Dermatology at Severance Hospital, Seoul, Republic of Korea, from December 2010 to July 2015, who were assigned to the external testing dataset. High-resolution (≥ 6 million pixels) digital cameras captured medical images of the anterior neck or axilla at the initial visit and 3, 6, and 12 months of follow-up. We additionally collected photographs of patients without scars in the anterior neck region at the same intuition as a control (‘normal’) group. Overall, 2724 images from 1043 patients were included in the main dataset, and 374 images from 240 patients were obtained from the external dataset (Supplementary Table S1).

### Data acquisition and preprocessing

Clinical data were collected for each patient visit, including age, sex, BMI, date after surgery (scar age), history of keloids, operation site, clinical scar characteristics (itching, pain, adhesion, tightening, induration, or edema), treatment sessions (initial visit and 3, 6, and 12 months of follow-up), and treatment response (for follow-up visits). The digital images of the anterior neck or axilla included in the study were de-identified and minimally cropped to contain adjacent anatomical structures around the scar; for example, we cut off the photos of the anterior neck to include the Adam’s apple to the sternal notch. In addition, each captured image was assigned a unique identifier and linked to corresponding clinical data. Importantly, when multiple images were taken from a single patient at different time points, each image was individually linked to two time-related factors (scar age and treatment sessions). Subsequently, these images were independently scored for scar severity by three board-certified dermatologists who specialized in scar treatment, using the VSS^17^. Based on the VSS score and the required scar treatment modalities, as judged by scar-specialized dermatologists who are board-certified and have more than five years of clinical experience in specialized scar laser clinics, we classified the scars into four categories according to their severity: normal, mild, moderate, and severe (Supplementary Fig. S1)^18^. When there was a unanimous agreement on the score for a specific image among the three evaluators, we adopted that score directly as the gold standard label. In cases where the voting results were divided, the professionals gathered, reviewed the image together for consensus, and assigned a single label. Treatment response was defined as a VSS score ≥ 50% or ≥ 2 decrements of severity grade compared with the initial visit.

### Neural network structure and training

We adopted the CBAM integrated with ResNet-50 for the image-based severity prediction model. CBAM consists of a channel and spatial attention submodules that focus on meaningful features and suppress unwanted ones^19^. In addition, an MLP model was trained to distinguish each severity class based on 11 collected clinical variables for clinical data-based severity prediction. Finally, the combined model for severity prediction was obtained from the 6:4 ratio of the weighted sum of the image-based and clinical data-based prediction models. Furthermore, we developed an image-based regression model to estimate the VSS based on the score of each image. The detailed processes and architecture of the AI model are described in Supplementary Text S1, Figs. S2 and S3.

### Evaluation of algorithm performance

The trained model was evaluated using the test datasets from the internal and external testing datasets. Next, the classification performance of the image-based severity prediction model was compared with the evaluations of eight board-certified dermatologists and eight dermatology residents. We randomly selected 240 images from the internal test dataset (60 images from each severity class), presented them as original resolution photographs, and asked the clinicians to select the most appropriate classification (single choice). A class activation map (Grad-CAM and Guided Grad-CAM), which allows the visualization of important features via gradient-based localization^20^, was implemented to qualitatively understand the prediction made by the deep network model. In addition, we examined the internal features learned by the models using t-SNE, which reduces the 2048-dimensional vectors obtained using the classification models to a 2-dimensional map.

### Statistical analysis

Five-fold stratified cross-validation was performed to verify the robustness of the best-fit model. The performance of each model was calculated using the Top-1 accuracy, sensitivity, specificity, and F1 score. ROC curves were drawn using sensitivity and specificity for each threshold, and AUCs were calculated. The 95% CIs were calculated using bootstrap resampling of the test dataset with the replacement N = 1000 times^21^. Categorical variables were compared using Fisher’s exact or chi-square tests with adjusted residuals if the variables were in 2 × 3 categorical tables. A one-way analysis of variance was used to compare continuous variables. Statistical analyses were performed using Python version 3.9.0, and *P* values < 0.05 were considered statistically significant.

### Ethic statement

The research adheres to the principles set forth in the Declaration of Helsinki and the ethical guidelines for human studies. The Institutional Review Board of Yonsei University Severance Hospital waived the need for informed consent from study participants due to the retrospective nature of the study (approval number 4-2022-0741). Nonetheless, explicit consent was acquired from the patient who consented to the publication of their clinical image as a figure in an online open-access journal.

## Results

### Patients and clinical characteristics

The study included a total of 1043 patients in the main dataset: 109 (10.5%), 705 (67.6%), and 229 (22.0%) had mild, moderate, and severe degrees of scar severity, respectively, according to the initial clinical presentation. When comparing the clinical variables between these severity groups, the following factors showed significant differences: BMI, date after surgery, minimally invasive thyroidectomy (MIT), modified radical neck dissection (MRND), transaxillary approach, itching/pain, adhesion/tightening, and induration/edema (Table S2).

To identify the predictive factors associated with scar severity, we performed multinomial logistic regression using the significant variables (*P* < 0.10) shown in Supplementary Table S1, with the moderate group as the reference group. In the multivariate model, MIT (odds ratio [OR]: 2.18, 95% confidence interval [CI]: 1.32‒3.60) and the date after surgery (OR: 1.04, 95% CI 1.03‒1.06) were positively correlated with mild scar severity. The transaxillary approach (OR: 3.11, 95% CI 1.75‒5.50), date after surgery (OR: 1.07, 95% CI 1.05‒1.09), and itching/pain (OR: 1.52, 95% CI 1.03‒2.24) were positively correlated with severe scar severity; however, adhesion/tightening (OR: 0.69, 95% CI 0.50‒0.97) and induration/edema (OR: 0.55, 95% CI 0.34‒0.89) were negatively associated with severe scarring (Table 1).

**Table 1:** Multinomial logistic regression analysis by scar severity groups.

Independent variables	Mild		Severe	
	OR (95% CI)	*P*-value	OR (95% CI)	*P*-value
Age at diagnosis	1.02 (0.99–1.04)	0.077	0.99 (0.98–1.01)	0.63
Body mass index (BMI)	0.97 (0.91–1.03)	0.34	1.04 (0.99–1.08)	0.064
Date after surgery (months)	1.04 (1.03–1.06)	< 0.001*	1.07 (1.05–1.09)	< 0.001*
Location of surgery				
Conventional	Ref		Ref	–
MIT	2.18 (1.32–3.60)	0.002*	0.69 (0.42–1.16)	0.16
MRND	0.41 (0.16–1.04)	0.061	1.31 (0.81–2.13)	0.27
Transaxillary	1.31 (0.51–3.36)	0.58	3.11 (1.75–5.50)	< 0.001*
Clinical scar characteristics				
Itching/pain	1.10 (0.63–1.92)	0.74	1.52 (1.03–2.24)	0.034*
Adhesion/tightening	1.10 (0.71–1.69)	0.67	0.69 (0.50–0.97)	0.032*
Induration/edema	0.65 (0.37–1.17)	0.15	0.55 (0.34–0.89)	0.014*

Individual effect sizes (OR) and 95% CI refer to the comparison of the mild and severe severity group with the moderate scar severity group as a reference for the outcome.

MIT, minimally invasive thyroidectomy; MRND, modified radical neck dissection; OR, Odds ratio; CI, confidence interval.

*Statistically significant P values (< 0.05).

### Model performance

**Figure 1:**
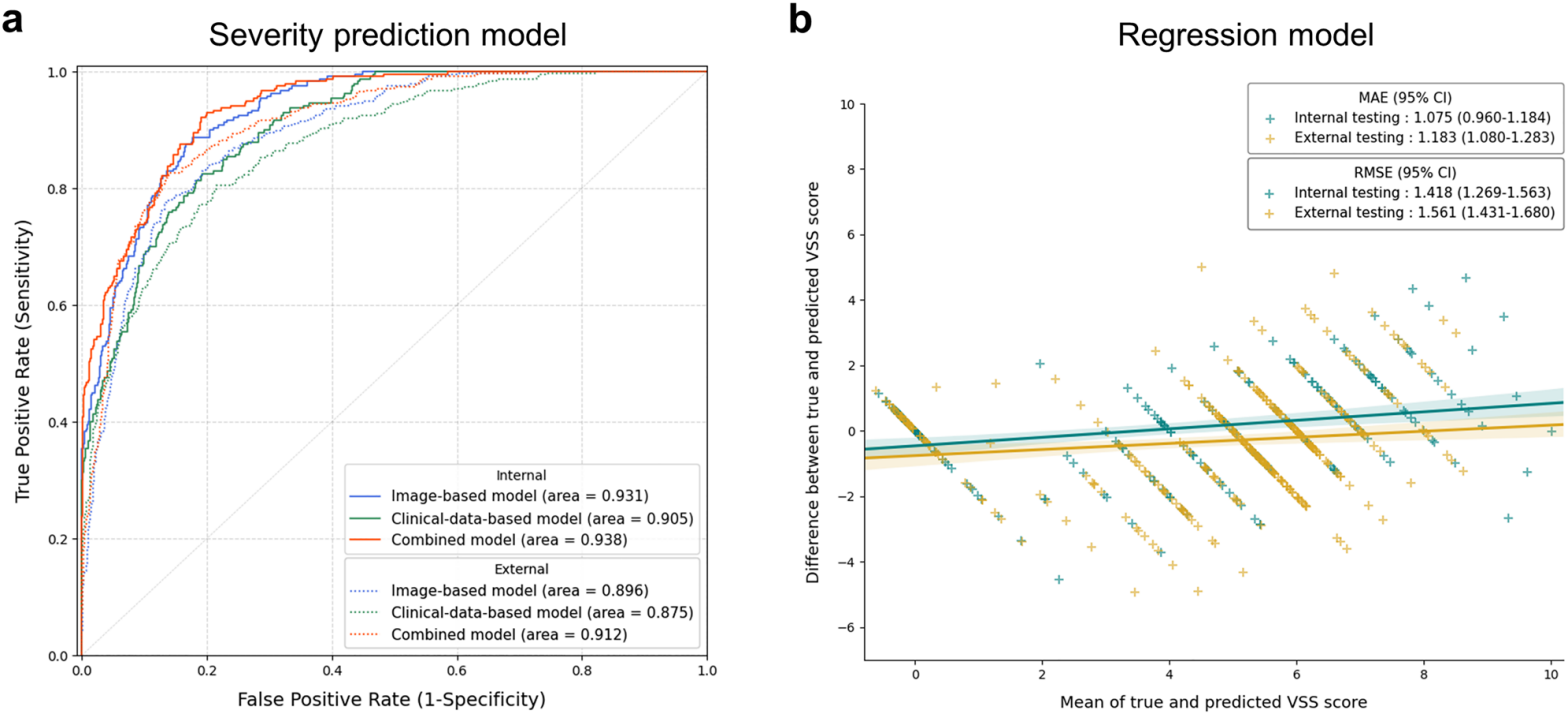
(**a**) Receiver operating characteristic (ROC) curves of the severity prediction models. Blue curve: image-based model by convolutional block attention module (CBAM) integrated Resnet-50, Green curve: clinical-data-based model by multilayer perceptron (MLP), Orange curve: combined model from the weighted sum of the image-based and clinical-data-based models. (**b**) Bland‒Altman plot shows the association between the measured and predicted Vancouver scar scale (VSS) score in the regression model. The shaded areas correspond to 95% confidence intervals. MAE; mean absolute error, RMSE; root mean square error.

We developed and validated three severity prediction models and one Vancouver scar scale (VSS) score regression model: (i) an image-based severity prediction model that integrated convolutional block attention module (CBAM) with CNN architecture, (ii) a clinical-data-based severity prediction model that used a multilayer perceptron (MLP) model with clinical variables, (iii) a combined severity prediction model derived from the weighted sum of models (i) and (ii), and (iv) an image-based regression model to predict the VSS score. The results for sensitivity, specificity, F1-score, receiver operating characteristic-area under the curve (ROC-AUC), and Top-1 accuracy of the severity prediction models are listed in Table 2. In the internal test dataset, the image-based model had a ROC-AUC of 0.931 (95% CI 0.910‒0.949), clinical data-based model had a ROC-AUC of 0.905 (95% CI 0.877‒0.928), and combination of these two models yielded a ROC-AUC of 0.938 (0.916‒0.955). In addition, the combined severity prediction model was significantly improved (*P* = 0·042) compared with the clinical data-based model; however, it was statistically insignificant compared with the image-based model (*P* = 0.633). Trends were similar in the external test dataset, yet slightly lower ROC-AUC and Top-1 accuracy were noted compared with the corresponding values in the internal test set (Fig. 1a). The sensitivity, specificity, F1-score, and ROC-AUC of each severity class in the internal and external testing sets are displayed in Supplementary Table S3.

**Table 2:** Performance of severity prediction models.

Model (class)	Sensitivity	Specificity	ROC-AUC	Accuracy	*P-*value^a^
	(95% CI)	(95% CI)	(95% CI)	(95% CI)	
Internal testing set					
Image-based model	0.725 (0.672–0.774)	0.908 (0.888–0.926)	0.931 (0.910–0.949)	0.725 (0.667–0.780)	0.633
Clinical data-based model	0.692 (0.638–0.750)	0.897 (0.879–0.917)	0.905 (0.877–0.928)	0.692 (0.638–0.750)	0.042
Combined model	0.730 (0.675–0.783)	0.910 (0.892–0.928)	0.938 (0.916–0.955)	0.729 (0.675–0.783)	Ref
External testing set					
Image-based model	0.695 (0.652–0.741)	0.898 (0.884–0.914)	0.896 (0.874–0.916)	0.695 (0.652–0.741)	0.260
Clinical data-based model	0.658 (0.610–0.706)	0.886 (0.870–0.902)	0.875 (0.848–0.899)	0.658 (0.610–0.706)	0.023
Combined model	0.733 (0.687–0.775)	0.911 (0.896–0.925)	0.912 (0.892–0.932)	0.733 (0.687–0.775)	Ref

ROC-AUC, area under the receiver operating characteristic curve; Ref, reference model; CI, confidence interval.

Calculated by the micro-averaged value of each severity class for the given model, using bootstrap resampling (N = 1000) of the test dataset.

^a^The P-value from the binomial test measures the difference in performance between the combined model and image- or clinical data-based model in terms of ROC-AUC.

The regression model for VSS score prediction utilized the mean absolute error (MAE), root mean square error (RMSE), and Bland‒Altman plot depicting the association between the predicted and measured VSS. The MAE of the internal testing set was 1.075 (95% CI 0.960‒1.184), and the RMSE was 1.418 (95% CI 1.269‒1.563). These values were slightly higher in the external testing set: 1.183 (95% CI 1.080‒1.283) for MAE and 1.561 (95% CI 1.431‒1.680) for RMSE. The Bland‒Altman plot showed a positive linear slope, indicating a positive proportional bias (Fig. 1b).

Five-fold stratified cross-validation was performed, and the Top-1 accuracy of the image-based and combined models fluctuated in the range of ± 1.6% and ± 4.0%, respectively, demonstrating the robustness of the models.

### Comparison between the neural network and dermatologists

**Figure 2:**
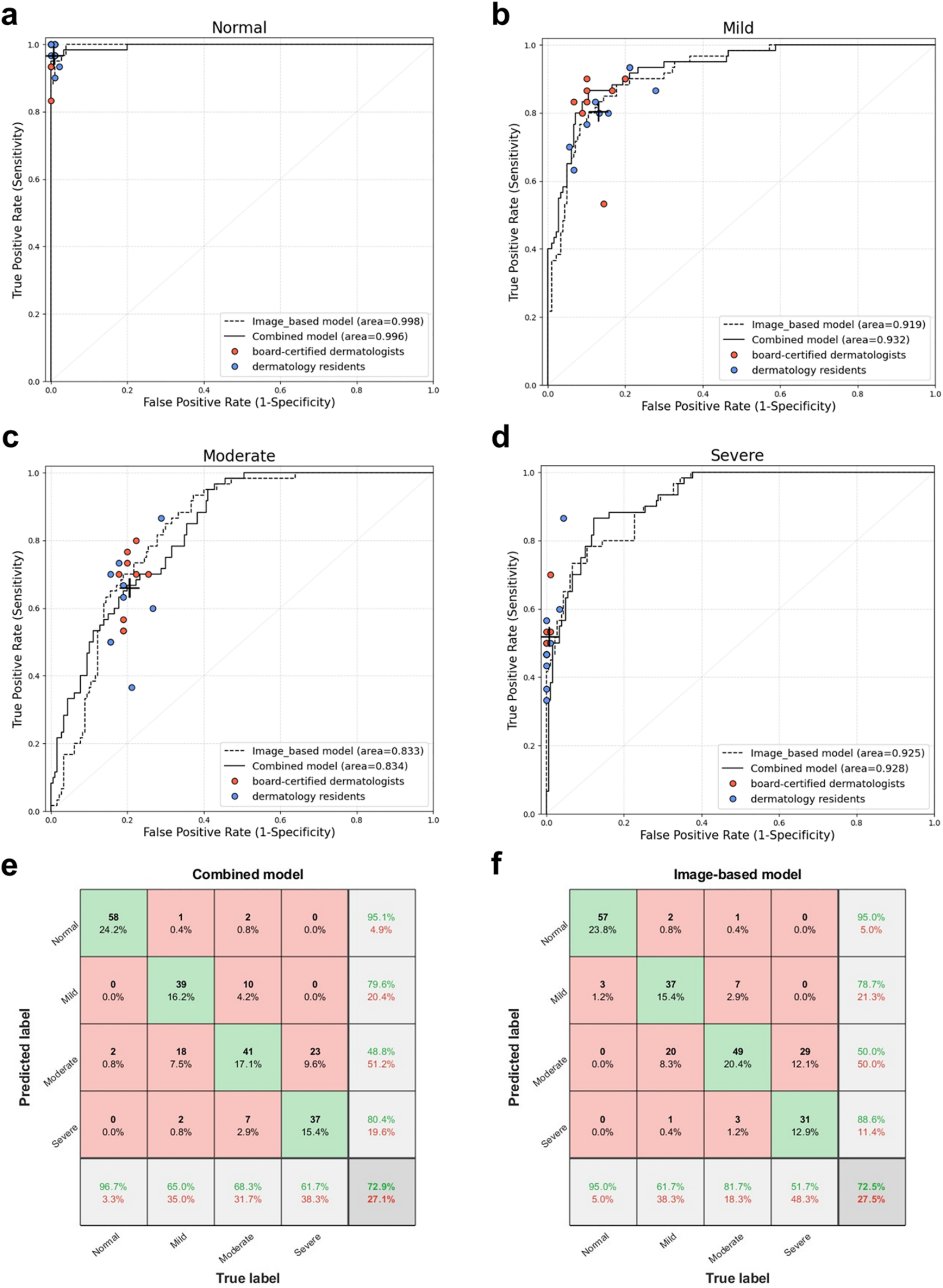
Scar severity classification performance of the convolutional neural network (CNN) and dermatologists. ROC (receiver operating characteristic) curves for each severity class were drawn for the image-based (dotted curve) and combined prediction model (black curve). In addition, the prediction value of the 16 dermatologists was plotted; Red dot = 8 board-certified dermatologists; Blue dot = 8 dermatology residents; Black cross = average value of 16 dermatologists. Performances for (**a**) Normal, (**b**) Mild, (**c**) Moderate, (**d**) Severe scars. (**e**) Confusion matrix of combined prediction model. (**f**) Confusion matrix of dermatologists.

We tested our model against eight board-certified dermatologists and eight dermatology residents to compare its performance. The overall Top-1 accuracies of the board-certified dermatologists and dermatology residents were 0.746 and 0.729, respectively. Image-based and combined models could classify four scar severity groups with a level of competence comparable with that of dermatologists (Fig. 2a–d). The confusion matrices of the neural network models and dermatologists over the four severity classes are shown in Fig. 2e and f. The AI models and dermatologists significantly confused mild and moderate scar lesions; the models had a slightly higher rate of misclassifying mild severity as moderate (7.5% *vs.* 4.3%), whereas humans had a higher rate of misclassifying moderate severity as mild (8.0% *vs.* 4.2%). In addition, both models and dermatologists tended to misclassify severe lesions as moderate (9.6% and 11.0%, respectively).

### Visualization of the explanatory model

**Figure 3:**
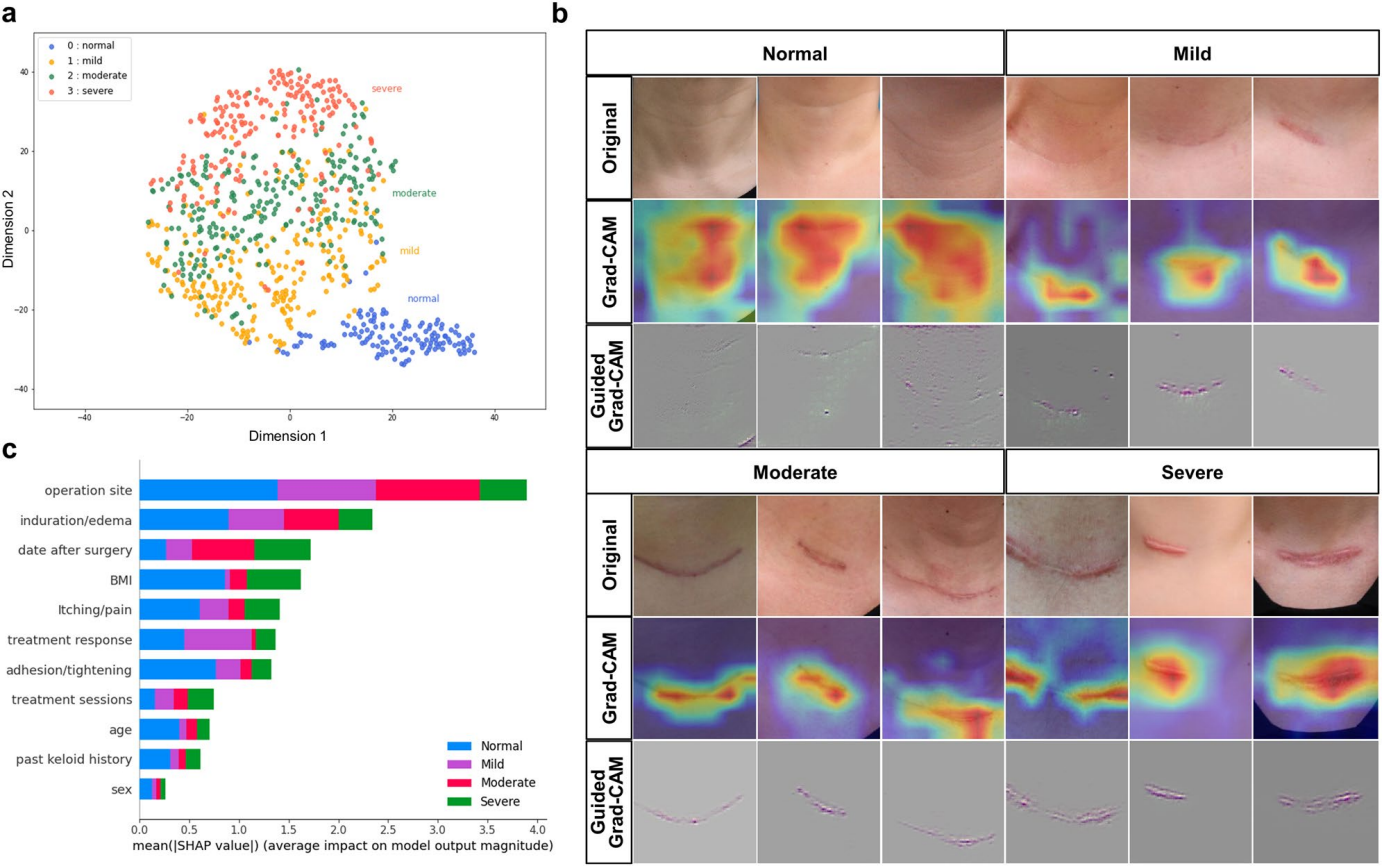
(**a**) t-distributed stochastic neighbor embedding (t-SNE) visualization of the last hidden layer representations in the image-based prediction model. The output of the neural network’s last hidden layer is projected onto a 2-dimensional map using the t-SNE method. Colored point clouds represent different severity classifications, showing how the algorithm clusters postoperative scars. (**b**) Visual explanations of postoperative scar cases via class activation mapping. Clinical images of each scar severity grade and corresponding heatmaps via gradient-based localization (Grad-CAM). The activation was focused on the hypertrophied region of the scar. (**c**) Interpretation of the clinical-data-based model via SHapley Additive exPlanations (SHAP) analysis. The importance ranking of variables used in the clinical-date-based model according to the mean (|SHAP value|).

We adopted two visualization methods for the image-based model: dimensionality reduction via t-distributed stochastic neighbor embedding (t-SNE), and Gradient-weighted Class Activation Mapping (Grad-CAM). Figure 3a shows the two-dimensional expression of the internal features extracted from the image-based classification model. The neural network model extracted distinct features for scar severity classification, and the cluster represented in each class occupied relative regions in the two-dimensional map corresponding to clinical features. For example, the mild class cluster is located between normal and moderate severity, and the moderate class is flanked by the mild and severe classes with some overlaps.

Figure 3b shows the results from the class activation mapping, in which the heatmaps represent the pixel areas activated by the deep neural network. The CBAM-integrated CNN model successfully distinguished postoperative scars from wrinkles in the surrounding skin. In addition, it could detect coarse and hypertrophic portions of the lesion in moderate or severe scars.

Furthermore, to elucidate significant variables in predicting the outcome of the clinical-data-based model, we introduced the SHapley Additive exPlanations (SHAP) method for visualizing the importance ranking of the features^22^. Figure 3c shows the importance ranking of all variables used in the clinical data-based model, evaluated by the average absolute SHAP value. Operation site, induration/edema, date after surgery, BMI, and itching/pain were the Top-5 dominating features for predicting scar severity.

## Discussion

All undesirable scars are unacceptable for different reasons^23^; thus, clinically, it is difficult to differentiate “undesirable” scars. Various scar assessment scales have been developed for clinicians to evaluate scar severity, progression, and treatment response; however, a “gold standard” scar scale is yet to be established^24^. Therefore, in this study, we aimed to evaluate postoperative scars using deep neural network models based on scar severity. Using AI models based on the patients' digital images and clinical information, we successfully classified postoperative scars according to their severity, and the performance of the models was comparable to those of dermatologists.

We intentionally collected and cropped digital images to include scars, adjacent skin structures, and artifacts such as clothes or rulers (Supplementary Fig. S1). Intensive preprocessing, including resizing and cropping the clinical image to include only lesions of interest for analyses, may help improve classification performance. However, this is a laborious and exhausting task far from the actual clinician’s viewpoint of scarring, which usually incorporates broader adjacent anatomical structures^6,25^. Thus, we integrated the CBAM into the CNN architecture, which selectively and automatically focuses on salient lesions, much like the human visual perception mechanism^19,26^. Therefore, our image-based model successfully classified scar severity while appropriately concentrating on the lesion of interest (Fig. 3b), without direct human labeling or cropping of the scar lesion.

To construct an image-based AI model, we classified postoperative scars into four subtypes based on the VSS, the first validated and most widely used scar scale to date^17^. The VSS consists of four parameters related to scar characteristics: height, pliability, pigmentation, and vascularity, to generate a semi-quantitative score ranging from 0 to 13 points^27^. However, the VSS has a significant limitation in that it does not reflect various factors that determine scar severity other than the morphological scar characteristics^17,24^. Therefore, we developed a neural network model trained with 11 clinical variables related to postoperative scars, including patients’ demographic features, symptoms, local complications, and scar age. The AI model based on clinical variables showed considerable performance in predicting the severity of postoperative scars; however, it was significantly lower than that with a combination of clinical variables and medical images. These results indicate the importance of utilizing scar-related clinical characteristics and morphological features when predicting the severity of the postoperative scar. Furthermore, we adopted the SHAP analysis to clarify the influential clinical features for predicting the severity of postoperative scars and provide a plausible interpretation of the model's decision-making process. The SHAP method took account of the most critical risk factors for postoperative hypertrophic scarring, including scar location, increased BMI, and subjective symptoms. These results correspond with those of previous multinomial logistic regression analysis and studies of postoperative scar risk factors^3,6,28^.

AI has performed at least equal or superior to dermatologists for diagnosing or classifying various skin diseases^8,9,29,30^. Our deep neural network model also showed performance comparable with board-certified dermatologists or dermatology residents in classifying postoperative scars according to their severity. We also need to consider the nature of the classification task in this study, which was not to distinguish different diseases but to grade the severity of the same disorder. Considering the semi-quantitative, rater-dependent, and subjective nature of the current scar-grading system^23^, significant ambiguity and overlap was expected between the classification classes used in this study. The confusion matrices revealed striking similarities in misclassification between humans and neural network models. The AI models and dermatologists tended to misclassify mild or severe scars as moderate. One plausible reason for this phenomenon is the insufficient distinctive features of intermediate-grade scars compared with other severity groups^31^; the other reason lies in the central tendency bias of visual perception, which is likely to estimate towards the mean of the stimuli^32^.

Our study has several limitations. First, the AI model showed decreased performance in the external testing set compared with that in the internal testing set. This could have been due to the different image acquisition settings of different hospitals. In addition, since the VSS has two components directly related to the color of the image (pigmentation and vascularity), slight differences in input in the color channels by individual camera settings may create substantial changes in the output of the model^33^. Second, due to the study’s retrospective design, data imbalance in the training dataset and possible selection bias may restrict the application of this study to the broader general population with postoperative scarring. In addition, several studies have assessed scar scales with a photograph-based examination by scar-specialized clinicians^23,34,35^; however, some VSS components (i.e., pliability or height) may be difficult to evaluate using only clinical images without examination of live scars. Last, our study cohort exclusively included Korean patients; hence, only patients with Fitzpatrick skin types III and IV were included in the dataset. Since darker skin type is one of the predisposing factors for hypertrophic scars^25^, future studies with larger-scale datasets from different ethnic groups with various scar etiologies are needed.

In conclusion, an AI model based on images and clinical data can predict the severity of postoperative scars. Our neural network models were trained with a relatively small (< 5000) number of images; however, they efficiently classified the severity of postoperative scar lesions with performance comparable with that of dermatologists. These models can aid clinicians in scar management to determine scar severity and make treatment decisions. We anticipate extending our established dataset of postoperative scars to other types of scars (such as burns, trauma, and post-infectious scars) in future studies.

### Supplementary Information

Supplementary Information.

## Data Availability

The data that support the findings of this study are available from the corresponding author upon reasonable request. Also, the relevant source code for developing and validating the neural network and all pixel-wise annotations were published in our public repository (https://github.com/dbssk6904/Scar-Severity-prediction-pytorch).
