# Supplementary material for: Predicting the severity of postoperative scars using artificial intelligence based on images and clinical data
Source: Sci Rep. 2023 Aug 18;13:13448. doi: 10.1038/s41598-023-40395-z (PMC10439171; doi:10.1038/s41598-023-40395-z)
Supplement: Supplementary file 1 — Supplementary Information. [file 41598_2023_40395_MOESM1_ESM.docx]

Predicting the Severity of Postoperative Scars Using Artificial Intelligence Based on Images and Clinical Data

Jemin Kim,^1,2#^ Inrok Oh,^3#^ Yun Na Lee,^4^ Joo Hee Lee,^4^ Young In Lee,^2,4^ Jihee Kim,^1,2^ Ju Hee Lee^2,4*^

^1^Department of Dermatology, Yongin Severance Hospital, Yonsei University College of Medicine, Gyeonggi-do, Korea

^2^Scar Laser and Plastic Surgery Center, Yonsei Cancer Hospital, Yonsei University College of Medicine

^3^LG Chem Ltd., Seoul, Korea

^4^Department of Dermatology and Cutaneous Biology Research Institute, Severance Hospital,
Yonsei University College of Medicine, Seoul, Korea

^#^These authors contributed equally to this work.

# SUPPLEMENTARY MATERIAL

**Supplementary text S1.** Model training

**Table S1.** Summary of the main and external dataset and corresponding demographic information

**Table S2.** Baseline patient characteristics and comparison of features stratified by initial scar severity

**Table S3.** Performance of the prediction model according to severity class in the internal and external testing sets

**Figure S1.** Description and representative images of scar severity classification according to morphological features and treatment requirements

**Figure S2.** Illustration of severity prediction model’s pipeline

**Supplementary text S1.** Model training

1-1) Training the image-based model

The convolutional block attention module (CBAM) has two sequential attention submodules: channel and spatial^1^. The channel attention module creates two descriptors via average- and max-pooling operations, which aggregate the spatial information from the feature maps. The two descriptors were element-wise summated through a shared network to create a channel attention map. The spatial attention module performs average- and max-pooling operations along the channel axis and concatenates each feature to create a descriptor, and applies a convolution layer to the created feature descriptor to create a spatial attention map. The attention map created from each attention module was multiplied by the input feature map to create refined features that focused on the meaningful information in the image.

To adjust the data imbalance between classes and avoid overfitting, data augmentation techniques, such as random image cropping and white balance, were adopted^2^. Further, a stratified K-fold cross-validation was utilized to maintain the proportionality of samples for each class across every fold, thus ensuring a more balanced training set. Additionally, for the evaluation of our model, we intentionally curated a internal testing set with an equal distribution of classes, adopting a 1:1:1:1 ratio across severity groups, to ensure fair and comprehensive assessment.

All images were resized to 224 × 224 pixels and normalized to a range of -1 to 1 for training. The number of input channels was changed to three for three RGB (red, green, and blue)-colored channel input images. The inputs from the previous layer were batch-normalized, and Relu was adopted as an activation function. The input image passed through ResNet-50 integrated with CBAM, became a tensor of size 1×1×2048, and passed through three additional fully connected dense layers (512, 64, and 4). The dropouts occurred at a rate of 0.5 for each dense layer except for the last layer. Cross-entropy loss was used to train the network, and the softmax operation was applied to the model output. A stochastic gradient descent (SGD) optimizer was used with a learning rate of 0.01 and batch size of 16. The learning rate dropped by 0.1-fold every 30 epochs. The detailed parameters of the image-based model are depicted as Figure S3a.

1-2) Training the clinical data-based model

Clinical data were oversampled to relieve data imbalance and mapped with augmented images in combination with the image-based model. In addition, continuous variables were rescaled using a standard scaler. The inputs from the previous layer were batch-normalized, and Relu was adopted as an activation function. The dropouts occurred at a rate of 0.5 for each hidden layer. Cross-entropy loss was used to train the network, and the softmax operation was applied to the model output. An SGD optimizer was used with a learning rate of 0.01 and batch size of 16. The learning rate dropped by 0.1-fold every 30 epochs. The detailed parameters of the clincal data-based model are summarized as Figure S3b.

1-3) Integration of the image-based and clinical data-based model

Building separate models based on image and clinical data and then fusing them to predict the severity of a disease has already been demonstrated in other research.^3^ The integration of these two models into a combined model means that each model's prediction weight values of scar severity are concatenated to be represented as a single predictive value. As shown in the figure below, the output logits from both the image-based and clinical-data-based models are multiplied by attention weight (0.6 and 0.4, respectively), then concatenated and processed through a fully connected layer and softmax to produce the final output of the combined model (Fig. S3c).

Determining attention weight is somewhat of a hyperparameter decision, in which researchers consider both clinical context and model performance. In our study, we followed the following steps to determine this ratio: First, we referred to recent scar assessment scales such as the modified VSS and POSAS, and noted that these scales typically incorporate scar descriptions and comorbidities in an approximate 8:2 ratio (Nguyen, Tuyet A., et al. Semin Cutan Med Surg 34, 28-36 (2015)). Since the clinical variables used in our research include demographics, patient history, time-related factors, and scar-related comorbidities, we determined that the attention weight ratio between the image and clinical models should be decided within a range from 5:5 to 8:2. We then conducted hyperparameter tuning by adjusting the attention weight within this range to find the most optimal performance for our model. This process resulted in the final selection of 6:4 (or 0.6, 0.4).

1-4) Computer settings

The process was conducted using a personal computer running Windows 10 with PyTorch deep learning framework version 1.9.0 (https://pytorch.org/) with CUDA 11.2/CuDNN 8.1.0 dependencies for graphics processing unit (GPU) acceleration (Nvidia, California). The system was equipped with an Intel i9-12900K 24-threaded 3.20-GHz computer processing unit (Intel, California), 32 GB DDR4 RAM, 1 TB SSD, and 2 units of NVIDIA GeForce RTX A4000 D6 16 GB GPU. The relevant source code for developing and validating the neural network and all pixel-wise annotations were published in our public repository (https://github.com/dbssk6904/Scar-Severity-prediction-pytorch).

**References**

1 Woo, S., Park, J., Lee, J.-Y. & Kweon, I. S. in *Proceedings of the European conference on computer vision (ECCV).* 3-19.

2 Takahashi, R., Matsubara, T. & Uehara, K. Data augmentation using random image cropping and patching for deep CNNs. *IEEE Transactions on Circuits and Systems for Video Technology* **30**, 2917-2931 (2019).

3 Jiao, Z. *et al.* Prognostication of patients with COVID-19 using artificial intelligence based on chest x-rays and clinical data: a retrospective study. *The Lancet Digital Health* **3**, e286-e294 (2021).

**Table S1.** Summary of the main and external dataset and corresponding demographic information

|  |  | **Dataset** | |
| --- | --- | --- | --- |
| **Characteristics** | | Main^a^ | External^b^ |
| Data collection period | | 2015. 9‒2021. 12 | 2010. 12‒2015. 7 |
| Dataset allocation | | Training (70%) Validation (10%) (Internal) Testing (20%) | External testing (100%) |
| Patient demographics | |  |  |
|  | Unique individuals, n | 1043 | 240 |
|  | Female sex, % | 88.7 | 87.9 |
|  | Age at diagnosis, mean ± SD | 40.5 ± 11.5 | 42.2 ± 11.2 |
| Number of images, n | | 2724 | 374 |
|  | Normal | 332 | 50 |
|  | Mild | 687 | 74 |
|  | Moderate | 1287 | 201 |
|  | Severe | 418 | 49 |
| Collection period of images, n | |  |  |
|  | Initial | 1354 | 290 |
|  | Post-3 months | 727 | 52 |
|  | Post-6 months | 434 | 28 |
|  | Post-12 months | 209 | 4 |

Abbreviations: SD; standard deviation

^a^Obtained from the Scar Laser and Plastic Surgery Center, Yonsei cancer hospital

^b^Obtained from the Department of Dermatology, Severance Hospital

**Table S2.** Baseline patient characteristics and comparison of features stratified by initial scar severity

|  |  | **Mild** | **Moderate** | **Severe** | **Total** | *P-*value |
| --- | --- | --- | --- | --- | --- | --- |
| **Feature** | | **(N=109)** | **(N=705)** | **(N=229)** | **(N=1043)** |  |
| Female sex | | 102 (93.6) | 625 (88.7) | 198 (86.5) | 925 (88.7) | 0.15 |
| Age at diagnosis | | 42.2 ± 12.1 | 40.6 ± 11.2 | 39.2 ± 12.2 | 40.5 ± 11.5 | 0.077 |
| Body mass index (BMI) | | 22.8 ± 3.50 | 23.2 ± 3.76 | 23.8 ± 4.36 | 23.3 ± 3.89 | 0.046^*^ |
| Date after surgery (months) | | 10.9 ± 18.9 | 4.14 ± 7.59 | 10.7 ± 13.8 | 6.29 ± 11.3 | <0.001^*^ |
| Past keloid history | | 1 (0.9) | 17 (2.4) | 8 (3.5) | 26 (2.5) | 0.38 |
| Radioiodine ablation | | 17 (15.6) | 172 (24.4) | 60 (26.2) | 249 (23.9) | 0.09 |
| Location of surgery | |  |  |  |  |  |
|  | Conventional | 68 (62.4) | 473 (67.1) | 149 (65.1) | 690 (66.2) | 0.59 |
|  | MIT | 29 (26.6)^†^ | 105 (14.9) | 24 (10.5) | 158 (15.1) | 0.001^*^ |
|  | MRND | 6 (5.5) | 92 (13.0) | 34 (14.8) | 132 (12.7) | 0.047^*^ |
|  | Transaxillary | 6 (5.5) | 34 (4.8) | 27 (11.8)^†^ | 67 (6.4) | 0.001^*^ |
| Clinical scar characteristics | |  |  |  |  |  |
|  | Itching/pain | 19 (17.4) | 123 (17.4) | 60 (26.2)^†^ | 202 (19.4) | 0.012^*^ |
|  | Adhesion/Tightening | 54 (49.5) | 346 (49.1)^†^ | 84 (36.7) | 484 (46.4) | 0.004^*^ |
|  | Induration/Edema | 17 (15.6) | 171 (24.3)^†^ | 26 (11.4) | 214 (20.5) | <0.001^*^ |

^*^Statistically significant P-values (<0.05)

^†^Statistically significant adjusted standardized residuals (>2.1)

Abbreviations: MIT, Minimally invasive thyroidectomy; MRND, modified radical neck dissection; BMI, body mass index.

**Table S3.** Performance of the prediction model according to severity class in the internal and external testing sets

|  |  |  | **Sensitivity** | **Specificity** | **F1-score** | **ROC-AUC** |
| --- | --- | --- | --- | --- | --- | --- |
| **Model (class)** | | | **(95% CI)** | **(95% CI)** | **(95% CI)** | **(95% CI)** |
| **Internal testing set** | | |  |  |  |  |
|  | Image-based model | |  |  |  |  |
|  |  | Normal | 0.950 (0.889‒1.000) | 0.983 (0.961‒1.000) | 0.950 (0.906‒0.984) | 0.998 (0.994‒0.999) |
|  |  | Mild | 0.617 (0.500‒0.729) | 0.944 (0.904‒0.976) | 0.692 (0.593‒0.781) | 0.919 (0.880‒0.951) |
|  |  | Moderate | 0.817 (0.712‒0.914) | 0.728 (0.667‒0.788) | 0.620 (0.525‒0.703) | 0.833 (0.781‒0.878) |
|  |  | Severe | 0.517 (0.390‒0.638) | 0.978 (0.954‒0.995) | 0.653 (0.530‒0.750) | 0.925 (0.884‒0.954) |
|  | Clinical-data-based model | |  |  |  |  |
|  |  | Normal | 1.000 (1.000-1.000) | 1.000 (1.000-1.000) | 1.000 (1.000-1.000) | 1.000 (0.999-1.000) |
|  |  | Mild | 0.817 (0.714-0.911) | 0.811 (0.757-0.865) | 0.685 (0.590-0.770) | 0.889 (0.840-0.928) |
|  |  | Moderate | 0.467 (0.333-0.593) | 0.900 (0.853-0.944) | 0.528 (0.404-0.644) | 0.808 (0.745-0.862) |
|  |  | Severe | 0.483 (0.362-0.614) | 0.878 (0.828-0.922) | 0.522 (0.409-0.632) | 0.805 (0.747-0.858) |
|  | Combined model | |  |  |  |  |
|  |  | Normal | 0.967 (0.918-1.000) | 0.983 (0.962-1.000) | 0.959 (0.917-0.991) | 0.996 (0.988-1.000) |
|  |  | Mild | 0.650 (0.518-0.762) | 0.944 (0.909-0.977) | 0.716 (0.608-0.797) | 0.932 (0.894-0.962) |
|  |  | Moderate | 0.683 (0.571-0.797) | 0.761 (0.699-0.823) | 0.569 (0.470-0.671) | 0.834 (0.781-0.882) |
|  |  | Severe | 0.617 (0.500-0.731) | 0.950 (0.914-0.979) | 0.698 (0.600-0.790) | 0.928 (0.895-0.959) |
| **External testing set** | | |  |  |  |  |
|  | Image-based model | |  |  |  |  |
|  |  | Normal | 0.980 (0.936-1.000) | 0.985 (0.970-0.997) | 0.942 (0.891-0.981) | 0.999 (0.997-1.000) |
|  |  | Mild | 0.284 (0.185-0.390) | 0.980 (0.962-0.994) | 0.416 (0.286-0.528) | 0.794 (0.733-0.849) |
|  |  | Moderate | 0.871 (0.822-0.918) | 0.549 (0.479-0.626) | 0.771 (0.731-0.810) | 0.764 (0.714-0.808) |
|  |  | Severe | 0.306 (0.179-0.451) | 0.923 (0.891-0.950) | 0.337 (0.206-0.463) | 0.704 (0.626-0.785) |
|  | Clinical-data-based model | |  |  |  |  |
|  |  | Normal | 1.000 (1.000-1.000) | 0.966 (0.944-0.985) | 0.901 (0.833-0.956) | 1.000 (0.999-1.000) |
|  |  | Mild | 0.514 (0.400-0.635) | 0.910 (0.873-0.942) | 0.547 (0.453-0.641) | 0.768 (0.700-0.837) |
|  |  | Moderate | 0.721 (0.657-0.781) | 0.653 (0.580-0.728) | 0.714 (0.661-0.763) | 0.764 (0.714-0.815) |
|  |  | Severe | 0.265 (0.150-0.396) | 0.908 (0.875-0.938) | 0.283 (0.161-0.400) | 0.631 (0.547-0.712) |
|  | Combined model | |  |  |  |  |
|  |  | Normal | 1.000 (1.000-1.000) | 0.988 (0.975-0.997) | 0.962 (0.920-0.991) | 0.999 (0.996-1.000) |
|  |  | Mild | 0.338 (0.239-0.200) | 0.997 (0.987-1.000) | 0.500 (0.381-0.612) | 0.833 (0.778-0.888) |
|  |  | Moderate | 0.915 (0.871-0.952) | 0.543 (0.467-0.615) | 0.793 (0.753-0.832) | 0.807 (0.761-0.854) |
|  |  | Severe | 0.306 (0.179-0.442) | 0.951 (0.926-0.972) | 0.375 (0.229-0.506) | 0.784 (0.714-0.849) |

ROC-AUC: receiever operating characteristic-area under the curve; CI: confidence interval

**Figure S1.** **Description and representative images of scar severity classification according to morphological features and treatment requirements.** The assessments by the three evaluators displayed high inter-rater consistency, as demonstrated by a Krippendorff's Alpha of 0.963 for the images of internal testing set.

*Abbreviations: VSS, Vancouver scar scale*

**
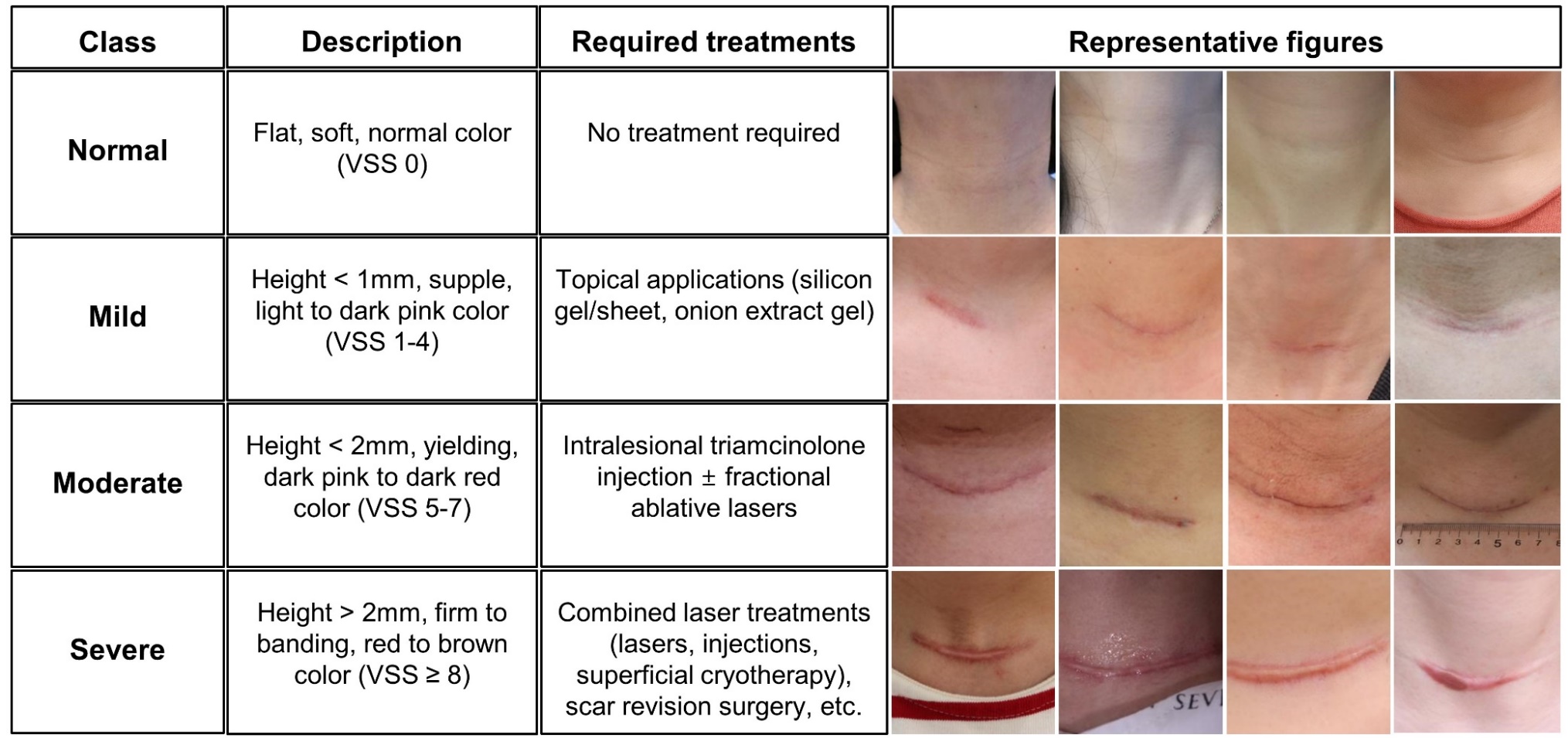
**

**Figure S2. Illustration of the severity prediction model’s pipeline**

*Abbreviations: CBAM, convolutional block attention module; CNN, convolutional neural network; MLP, multilayer perceptron*

**
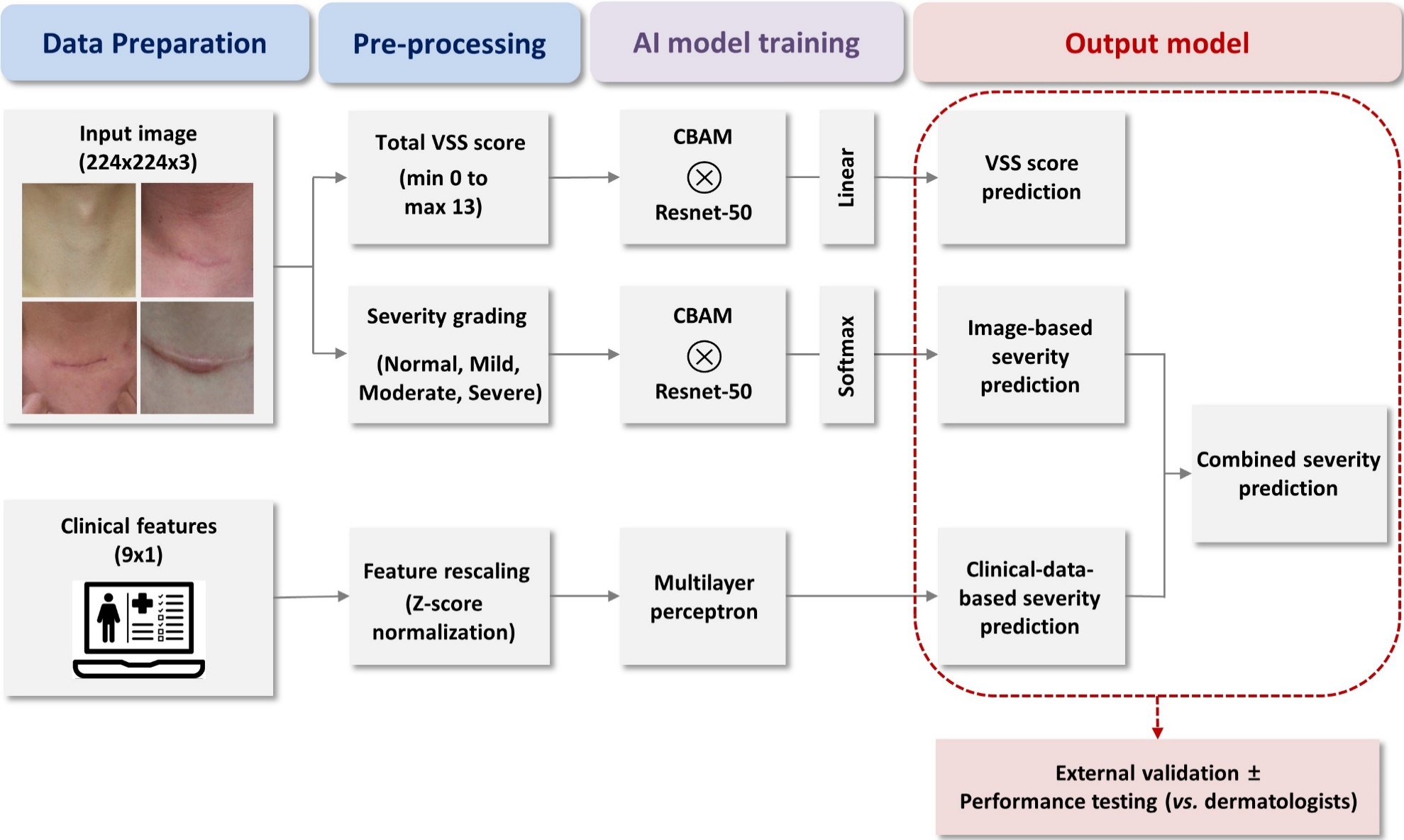
**

**Figure S3. Detailed architecture of the AI model.** (a) Detailed parameters of the image-based model. (b) Detailed parameters of the clinical-data-based model. (c) Integration of the image-based and clinical data-based model

*Abbreviations: CBAM, convolutional block attention module; CNN, convolutional neural network; MLP, multilayer perceptron; FC, fully-connected*

*
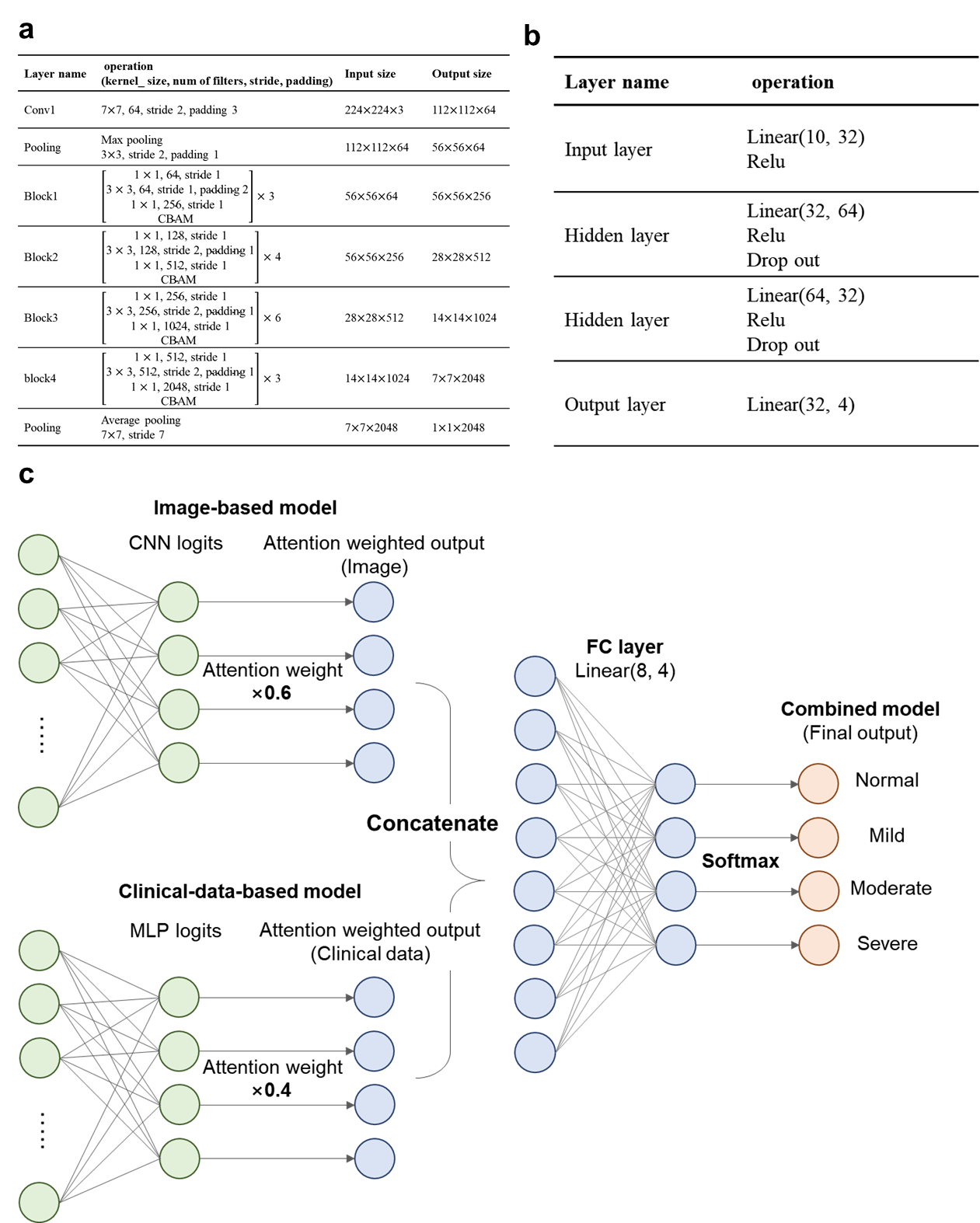
*
